# Perspectives on the Combined Use of Electric Brain Stimulation and Perceptual Learning in Vision

**DOI:** 10.3390/vision6020033

**Published:** 2022-06-14

**Authors:** Marcello Maniglia

**Affiliations:** Department of Psychology, University of California at Riverside, Riverside, CA 92507, USA; mmanig@ucr.edu

**Keywords:** perceptual learning, brain stimulation, visual training, clinical applications

## Abstract

A growing body of literature offers exciting perspectives on the use of brain stimulation to boost training-related perceptual improvements in humans. Recent studies suggest that combining visual perceptual learning (VPL) training with concomitant transcranial electric stimulation (tES) leads to learning rate and generalization effects larger than each technique used individually. Both VPL and tES have been used to induce neural plasticity in brain regions involved in visual perception, leading to long-lasting visual function improvements. Despite being more than a century old, only recently have these techniques been combined in the same paradigm to further improve visual performance in humans. Nonetheless, promising evidence in healthy participants and in clinical population suggests that the best could still be yet to come for the combined use of VPL and tES. In the first part of this perspective piece, we briefly discuss the history, the characteristics, the results and the possible mechanisms behind each technique and their combined effect. In the second part, we discuss relevant aspects concerning the use of these techniques and propose a perspective concerning the combined use of electric brain stimulation and perceptual learning in the visual system, closing with some open questions on the topic.

## 1. Introduction

The idea of improving one’s eyesight, hearing or tactile perception has fascinated humans for centuries. In this perspective paper, we discuss the combined use of two techniques, perceptual learning and brain stimulation, whose individual use dates back more than 100 years ago. Perceptual improvements through repeated practice was first reported in scientific papers in the second half of the XIX century [1], while the use of devices to deliver electric stimulation to the brain as a means to improve perceptual functions has been reported as early as the XVIII century [2]. Both these techniques went on to greatly contribute to our understanding of the mechanisms through which our brain responds, learns, and adapts to sensory stimulation.

### 1.1. Improvements through Practice

**Figure 1:**
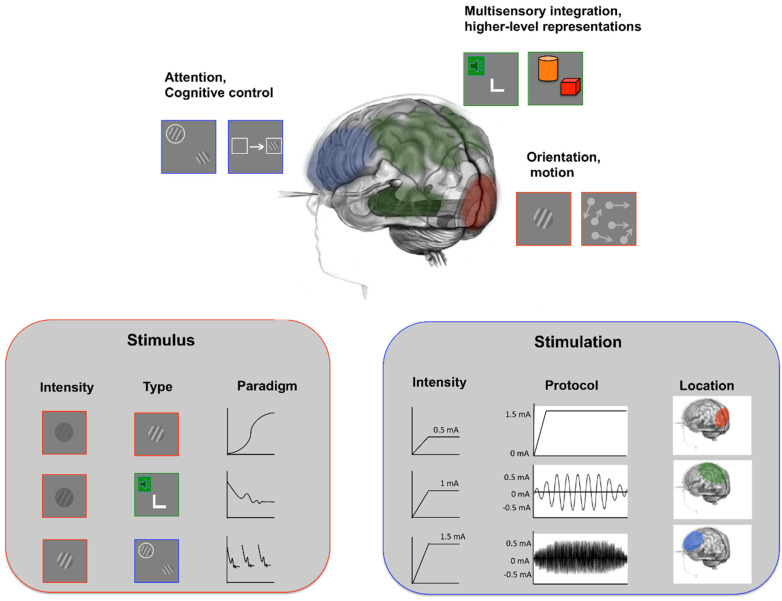
**Stimulation and training features contributing to learning outcomes.** Our current understanding of both VPL and tES indicates that several elements concur in generating the behavioral improvements reported in both literature studies, and optimizing these aspects can help boost learning outcomes. Characteristics of the visual stimulus can engage different sensory, attentional and cognitive areas, while characteristics of the stimulation can boost or inhibit perceptual and learning outcomes.

Behavioral studies investigating practice-induced sensory improvements commonly fall under the name of perceptual learning [3]. Over the last decades, perceptual learning has been applied to every sensory domain, building a rich literature that includes numerous models [4,5,6], proposed mechanisms [7,8,9,10], and sophisticated paradigms [11,12,13]. In the visual domain, visual perceptual learning (VPL) effects have been observed for a large variety of tasks, such as contrast detection [14,15,16], motion perception [17,18,19], visual search [20,21,22], texture discrimination [23,24], and more. Foundational VPL studies suggested that the observed improvements following extensive practice were due to neural plasticity changes at the earlier stages of sensory processing [23,25] that could in turn lead to improvements at later stages of perception, relying on input signals originating from these early areas [26]. In this context, VPL-mediated improvements were understood to be limited by the properties of neurons at the early stages of visual perception, thus leading to learning gains specific for stimulus features such as orientation, spatial frequency, retinal location, and the eye [23,24,27,28]. However, more recent studies and models depict a rather complex scenario, in which multiple mechanisms, processes and brain regions can be involved, in series or in parallel, in giving rise to learning [8,11,29,30,31] (see Stimulus box in Figure 1). A better understanding of VPL mechanisms led to the realization that features earlier reported, such as learning specificity, might be consequences of tasks and stimuli used in classic VPL studies rather than defining characteristics of it [6]. Crucially, generalization of learning from a limited set of stimuli to everyday life visual needs would improve VPL’s translational value, opening up possibilities for its use in visual rehabilitation and clinical interventions [32,33]. There is evidence of positive VPL results in treating some clinical pathologies such as myopia [34], presbyopia [26], and amblyopia [35], although the results are slightly less encouraging for more serious visual pathologies such as central vision loss due to macular degeneration [33,36,37,38].

### 1.2. Improvements through Electric Brain Stimulation

The last few years have witnessed an upsurge in studies describing the effects of different types of brain stimulation on modulating brain cortical activity and enhancing sensory performances [39,40] (see Stimulation box in Figure 1). Different types of non-invasive brain simulation techniques fall under the umbrella term of transcranial electric stimulation (tES) [41]. 

*Direct current*: A form of tES that has found large use in vision sciences is transcranial Direct Current Stimulation (tDCS) [42,43,44,45,46]. In this protocol, a weak electric current (1–2 mA) passes through two electrodes placed on the participant’s scalp, referred to as anodal (+) and cathodal (-) electrodes. This current is able to modulate the frequency rate of firing of the neural populations underneath by inducing polarity-specific changes in neural excitability [45]. Unlike other brain stimulation techniques such as transcranial magnetic stimulation (TMS) or deep brain stimulation (DBS), the intensity of the current used for tES is not sufficient to induce neuronal activation per se, but can cause alterations of resting membrane potential [47]. The effects of tES have been initially studied in the motor cortex, where they are well characterized. Specifically, anodal tDCS increases and cathodal tDCS decreases cortical excitability (as measured by TMS-induced motor-evoked potential (MEP) thresholds [45,46,48,49,50]). Furthermore, anodal tDCS over the motor cortex on five consecutive days during motor task sessions significantly enhanced the learning of the complex motor skill task when compared to a no stimulation (sham) condition [51]. 

Conversely, tES use in the visual cortex has a more recent history [52,53,54,55,56,57,58] and shows a higher degree of variability. While there is evidence of increases in visual cortex excitability via anodal tDCS and decreases via cathodal tDCS [43,44,52], other studies found conflicting results in visual-evoked potential (VEP) component modulation. Anodal stimulation increased and cathodal stimulation decreased the amplitude of the N70 component [59], while the opposite has been observed for the P100 component [52]. The authors in [60] showed that anodal tDCS transiently and significantly increased VEP amplitudes and contrast sensitivity (CS) in amblyopic eyes, while cathodal tDCS decreased VEP amplitude and CS. However, some other studies failed to observe any amplitude modulation after tDCS [61,62]. Behaviorally, Antal and colleagues [53] reported significant decreases in static and dynamic CS after cathodal stimulation, whereas anodal stimulation did not produce significant CS changes, a result corroborated by Chaieb and colleagues [55]. Conversely, other studies reported significant effects of anodal stimulation on CS, in particular for high spatial frequencies in healthy participants [54,63,64,65] and individuals suffering from amblyopia [58] and no effect of cathodal stimulation [63]. Reinhart and colleagues [64] showed that anodal stimulation improved Vernier acuity and increased VEPs amplitude, while cathodal stimulation had the opposite effects on both. Effects of anodal tDCS have also been reported to reduce surround suppression [66].

*Alternate current:* Alongside anodal and cathodal tDCS, the two other most common tES techniques seem to be transcranial alternating current stimulation (tACS) and transcranial random noise stimulation (tRNS), in which the current flow direction, and consequently the role of the electrodes, change over time. Specifically, during tACS, the current is delivered with an alternated sinusoidal pattern at a single specific frequency, while during tRNS, the current is delivered in a range of frequencies (usually between 0.1 and 1000 Hz), although the current is often referred to as hf-tRNS for frequencies starting from above 600 Hz and lf-tRNS for frequencies below 100 Hz, see [57,67]. Both protocols have been successfully used to boost visual functions such as CS [54], crowding [68], motion discrimination [69], and orientation discrimination [57].

There is evidence of tES-related behavioral effects on visual tasks for stimulation of areas beyond the early visual cortex. Olma and colleagues [70] observed improvement in motion perception, a function attributed to the extrastriate area MT, after occipital anodal tDCS, while Battaglini and colleagues [68] reported improvements in visual crowding reduction following parietal cortex stimulation.

## 2. Perceptual Learning Combined with Different Types of tES

As mentioned earlier, VPL can improve sensory processing and in turn perceptual abilities. These improvements, however, often come with some constraints. For example, the majority of classic perceptual learning studies reported high degrees of learning specificity (i.e., lack of transfer of training effects to different tasks, stimuli, retinal location, etc.) and a large number of training sessions/hours of practice necessary to observe significant results [3] (however, see [71]).

As a first step toward understanding whether electric brain stimulation can alleviate some of these constraints, Fertonani, Pirulli and Miniussi [57] compared the effects of different brain stimulation protocols, specifically anodal tDCS, cathodal tDCS and tRNS, while participants were engaged in an orientation discrimination task. Results showed significant between-blocks improvements when electric brain stimulation, specifically anodal tDCS and tRNS, was delivered on the occipital cortex of participants when compared to sham stimulation (control condition). Additionally, the authors reported larger learning rates for tRNS with respect to anodal tDCS, as the stimulation protocol was coupled with training. Further studies using anodal tDCS and tRNS showed their effectiveness in promoting visual functions in healthy participants, both in fovea when paired with orientation and contrast detection tasks [72,73] and in peripheral vision when paired with crowding reduction training [74].

A series of studies have used tACS in combination with PL to benefit from tACS’s ability to transiently modulate brain oscillations at specific frequencies [75,76,77,78] some of which, i.e., those in the alpha band (8–12 Hz), are associated with learning and consolidation [79]. He and colleagues [80] showed that occipital tACS at 10 Hz, but not at 20 Hz or 40 Hz, increased both learning rate and performance improvement during an orientation discrimination task. Crucially, tES appears to boost both the early (within session [57]) and late (between sessions/days [81,82]) components of VPL, and its behavioral effects seem to be long-lasting [83,84] (however, see [74]). There is also evidence that tRNS coupled with training induces larger transfer of learning with respect to behavioral training or brain stimulation alone [82,85].

Taken together, results from this literature study suggests that combining electric brain stimulation and VPL can boost the learning rate of a task and reduce the time/number of sessions/trials needed to observe significant training improvements.

Importantly, behavioral effects of electric brain stimulation and perceptual learning on visual tasks can be observed for targeted regions beyond occipital, sensory areas. Contò and colleagues [86] applied tRNS over the intraparietal sulci during attentional training and found behavioral improvements and an increase in resting-state functional connectivity within the dorsal and ventral attention networks. This opens up the possibility to use brain stimulation to modulate several aspects of learning beyond low-level sensory enhancement, including, but not limited to, attention, oculomotor control, expectations, task and instruction comprehension [6].

## 3. Time Course of Different tES Protocols

A crucial aspect of tES, both when applied by itself and when combined with VPL, is its effect in relation to the time of delivery. The interaction between timing of stimulation and learning has been initially explored in the context of motor learning [87,88,89], with studies showing that anodal tDCS increased the rate of learning when applied ‘online’ (during the execution of the task) [88,89] but not ‘offline’ (before the task) [89]. Conversely, in the visual domain, Pirulli, Fertonani and Miniussi [72] reported significant improvement in performance when anodal tDCS was applied offline but not when it was applied during training, while the opposite was observed for tRNS. The evidence that the same tES protocol led to different behavioral outcomes suggests that the neural modulation effects induced by tES differ depending on the excitability levels of the stimulated neurons at the time of stimulation application [72]. Other studies showed that the time interval between consecutive stimulation periods influences the neural modulation effect. Fricke and colleagues [90] showed that 5 min of anodal tDCS increased neuronal excitability for approximately 5 min; however, when a second 5 min anodal tDCS period follows after 3 min from the end of the previous session, the second session has the opposite effect, thus leading to a decrease in cortical excitability. Similarly, Monte-Silva and collaborators [91] showed that a second period of stimulation during the after-effects of a first period initially causes a decrease in motor cortex excitability, which then turns into an increase in excitability. While Pirulli, Fertonani and Miniussi [72] reported offline effects of anodal tDCS when it was delivered before training on orientation discrimination, Yang, He and Fang [73] more recently showed that anodal tDCS delivered after the training session, during a texture discrimination task, led to larger learning effects than the sham, suggesting that anodal tDCS might help consolidate learning across sessions, consistent with what was observed in the motor learning [51].

Additionally, there is evidence that cortical modulation effects induced by tES extend beyond the stimulation window [47], possibly involving long-term potentiation-like mechanisms [92]. Kasten, Dowsett and Herrmann [93] reported a sustained enhancement of alpha power 70 min after occipital tACS at individual alpha frequency. Similarly, occipital tRNS exhibited consistent excitability (decrease in phosphene threshold), lasting 60 min post-stimulation [94], similar to what was reported for motor cortex stimulation [56].

## 4. Mechanisms of tES

The effectiveness of tES, alone or when paired with training, has often led, especially early on, to an overlook of the mechanisms involved in the observed phenomena, although the recent literature has offered an excellent overview of mechanisms and models (e.g., [41,95]). The exact mechanisms governing tES effects are still elusive, and animal models are lacking; thus, hypotheses on the mechanisms in the human brain are speculative. Concerning tDCS, one of the most common interpretations of its effects is that the weak current that travels from one electrode (anode) to another (cathode) induces polarity-specific effects on the cortical excitability of regions traversed by the current. Specifically, regions under cathodal stimulation experience membrane hyperpolarization, which has been shown to decrease cerebral excitability, while conversely, regions under anodal stimulation undergo depolarization, which leads to increased cortical excitability. However, prolonged delivery of anodal tDCS might cause sustained depolarization [96]; thus, an initial facilitatory effect might reverse into inhibition in case of longer stimulation periods. These saturation mechanisms may not occur when the stimulation is delivered before the task [72].

It has been suggested that anodal and cathodal tDCS might modulate distinct neuronal populations. Recent studies showed that anodal tDCS reduces intracortical inhibition [89,97] through a reduction in GABA concentration [98,99,100,101,102]. GABA-mediated inhibition has been linked to a number of suppressive neural interactions within the visual cortex such as those underlying surround suppression [103,104]. Consistently, Spiegel and collaborators [66] observed significant reductions in psychophysically measured surround suppression following anodal tDCS. GABA-mediated inhibition is one of the proposed mechanisms regulating cortical plasticity in rodent models of deprivation amblyopia [105], and behavioral results in humans show CS improvements in adults with amblyopia [60]. Moreover, cortical inhibition mediated by GABA seems to cast a constraint on brain plasticity, especially in the visual cortex [106,107,108,109], and some visual pathologies such as amblyopia appear related with abnormally higher cortical inhibition. A previous study has shown that inhibiting monoamine reuptake enhances the duration of the aftereffects of anodal tDCS, and that both anodal and cathodal after-effects were reduced by a β-adrenergic receptor blocker [110]. This is consistent with the suggestion that tDCS might ‘substitute’ neuromodulatory effects normally associated with neurobiological systems, in particular that of the norepinephrine (NE) circuit [111].

Conversely, cathodal tDCS does not seem to affect GABA-mediated inhibitory interactions. For example, administering a GABA-antagonist does not affect the reduction of motor cortex excitability induced by cathodal tDCS [112], while it blocked the reduction of intracortical inhibition normally observed with anodal tDCS. This is consistent with behavioral data showing no effect of cathodal tDCS on surround suppression [66]. By contrast, Stagg and colleagues [101] observed modulation of glutamate levels following cathodal tDCS. A functional MRI study also suggested that anodal and cathodal stimulations modulate distinct systems-level networks within the active motor system [113].

Regarding tACS, evidence suggests that the alternated current, delivered at a specific frequency, can entrain cortical oscillations [78,114]. A possible alternative interpretation is that tACS might induce long-lasting plasticity-like changes, which have been observed both in the motor [115] and the visual cortex [116].

Concerning tRNS, Terney and colleagues [56] suggested that the repeated stimulation of tRNS might allow Ca2+ and Na+ channels to rapidly reopen [117]. This repeated subthreshold stimulation could induce an increase in the sodium inflow and a consequent prolonged depolarization and induction of long-term potentiation-like phenomena [56,57,117]. Pirulli, Fertonani and Miniussi [72] suggested that the larger effects observed for online tRNS with respect to offline anodal tDCS on learning rate might be explained by temporal properties of this protocol and its interaction with task-induced activity. The repetitive and random-wave shape of tRNS would lead to temporal summation of stimulus-induced activity, thus boosting neural activity in the process. The authors further suggested that the high frequency of tRNS (600–1000 Hz) may interact optimally with neural activity because it approaches the time constant of the cell body and dendrites, between 1 and 10 ms [118]. Conversely, if the stimulated neural population is not involved in the task execution, no task-related neuronal activity should occur, and the effects of tRNS should be null, which is consistent with experimental data with offline tRNS [57].

In the context of tES effects on the visual system, a model proposed by Miniussi and colleagues addresses the interaction between stimulation intensity and cortical activity elicited by a visual stimulus. This stochastic resonance framework of brain stimulation [119] combines the interaction between external noise coming from the tES, internal baseline activity and stimulus-driven activity, providing predictions on the perceptual outcome. In particular, in case of a low intensity stimulus, external noise would enhance the signal above the perceptual threshold, increasing the signal-to-noise ratio; while in case of a higher intensity stimulus, the external noise would disruptively boost the non-signal spontaneous internal activity, thus reducing the signal-to-noise ratio [120,121].

## 5. Perceptual Learning, tES and Clinical Populations

As mentioned earlier, VPL has been used in pathologies in which the optical or cortical aberrations are relatively mild and can be counteracted by neural plasticity to achieve total restoration of foveal functions (e.g., [26,34,35]). VPL has the potential of becoming the treatment of election for treating sensory pathologies [32,33]; however, some of its drawbacks, in particular the large number of sessions required to observe significant results and the high degree of learning specificity often reported, still limit its large-scale use in clinical populations. Adding tES to VPL holds the promise of addressing such limitations. There is evidence of larger training effects [57,74] and transfer [81,82,122] when electric brain stimulation was coupled with VPL with respect to VPL alone. Specifically, Camilleri and colleagues [81] used VPL in combination with tRNS in myopic patients. The results showed that 2 weeks of training with this protocol led to improvements in visual acuity (VA) comparable to those observed after 8 weeks with a purely behavioral training, with additional improvement in CS that was not observed in the behavioral-only training.

Similarly, Campana and collaborators [82] showed that the combination of tRNS and contrast detection training substantially improved VA and CS of amblyopic patients after eight sessions of training, with effect sizes comparable to those reported in a previous behavioral study with 30 to 80 training sessions [26]. More recently, Herpich and colleagues [84] used tDCS and tRNS coupled with VPL to train cortical blindness (CB) patients on motion perception in their blind field for 10 days. The results showed fast and long-lasting (6-month follow up) improvements in motion perception for the three CB patients in the tRNS group, while tDCS showed no advantage over the training-only group and the control region stimulation group.

## 6. Considerations on the Use of tES with VPL

tES and VPL are both valuable tools for inducing performance-enhancing neural plasticity. With the right understanding of their combined use, the outcome could be extremely valuable, from a theoretical and clinical perspective. However, both fields present complexities that are often overlooked, while conflicting results are sometimes left unaddressed. For example, while some of the disagreements between motor and visual tES studies may be explained by structural [123,124] and functional [125,126] differences between neural regions, the observation opens a larger point on which elements concur in producing the tES effects observed in different studies. The fact that multiple aspects most likely concur in the effects reported by brain stimulation studies has been acknowledged by several authors (e.g., [41,95,127]). Similarly, several authors in the field of PL are working on new and more refined models to describe the mechanisms governing learning and plasticity and to address relatively recent evidence in apparent contradiction with earlier (and simpler) models (e.g., [11,13]). We here identify four aspects that we consider crucial in the understanding behavioral effects of tES and PL.

Anatomy. As mentioned earlier, anatomical and functional characteristics of the stimulated area play a role in the brain stimulation effects. For example, stronger occipital tES effects would be expected for higher spatial frequencies because the current is most intense the closest to the surface of the cortex [128,129], where V1 neurons are tuned toward higher spatial frequencies [130,131]. Similarly, neurons located away from the occipital poles have peripheral receptive fields; thus, stimuli presented in the parafovea might not be affected as strongly by tES with respect to foveal stimuli [63]. Furthermore, the orientation of the stimulated units might play a role in the effect of tDCS [95], with evidence showing that neurons with axons parallel to the electric field are activated by anodal and are inhibited by cathodal tDCS [47,132], while the opposite has been observed for non-apical dendrites [133]. Finally, the spatial extent of tDCS-induced cortical modulation can reach beyond the targeted regions to structures connected to them [134].

Baseline performance. Sensory systems are ‘tuned’ to preferred features of the sensory stimulation, which determine the baseline performance of the system and in turn modulate tES effects. For example the visual system has higher sensitivity for stimuli oriented along cardinal orientations [135,136], thus potentially reducing room tES-mediated improvements in sensitivity along those axes, but allowing room for tES inhibitory effects (i.e., cathodal tDCS). Conversely, oblique stimuli may be more responsive to the facilitatory effects of anodal tDCS [65]. In the context of cognitive and memory studies, it has been shown that the effect of the stimulation correlates with the baseline performance of the participant [137,138].

Stimulus intensity and type. Evidence from perceptual studies show that the type of stimulus can selectively engage different cortical regions, while occipital tDCS studies show opposite phase-dependent modulation of the same VEP component when participants were tested using different stimuli (checkerboard pattern-reversal in [52] vs. stripe pattern-onset in [43]), thus suggesting an equally relevant role of stimulus type in both contexts. Similarly, Antal and colleagues [59] showed that cathodal tDCS over the occipital cortex improved or impaired motion perception depending on the type of motion stimulus.

Stimulus intensity affects the perceptual outcome by modifying the signal-to-noise ratio between the target and distractors/background. Consequently, low stimulus signal might be enhanced by tES, while high signal might be disturbed [119]. In the context of VPL, it then becomes important to calculate performance level across training days to estimate the optimal signal intensity. Studies utilizing low signal-to-noise perceptual threshold tasks detected significant anodal tDCS effects on CS [63,139], while a similar study identified no stimulation effects of anodal tDCS when supra-threshold stimuli were used [53]. Similarly, Antal and collaborators [43] reported phase-dependent modulation of N70 component amplitude with occipital cathodal and anodal stimulation, but only with presentation of low contrast stimuli. Higher contrast stimuli might lead to optimal activation of visual areas responding to the stimuli, such that the subthreshold (excitatory) modulation effect of the tDCS would not be able to modify the VEP amplitude. Stimulus intensity also seems crucial in eliciting transfer of learning, with training with para-threshold stimuli usually leading to better learning and transfer effects with respect to supra-threshold stimuli in healthy participants [3,140,141] and transfer [26] as well as in some clinical populations [142].

Stimulation intensity and type. Mirroring the previous point, intensity and type play a crucial role for stimulation as well. Changes in stimulation intensity can give rise to opposite outcomes for identical stimulation protocols. Pavan and colleagues [67] showed that tRNS modulated performance in a global motion perception task as a function of the stimulus intensity, with low (1.5 mA) stimulation leading to optimal performance and high (2.5 mA) stimulation leading to impairment of motion discrimination, while evidence from tACS studies at 140 Hz suggests that the excitatory effects observed at 1 mA [143] might be reversed at lower (0.4 mA) stimulation intensities [144]. Some studies reported an increase in cortical excitability proportional to stimulation intensity in the motor cortex [145], while others reported a less straightforward relationship [146].

Similarly, stimulation type can lead to different outcomes, with anodal tDCS generally enhancing and cathodal tDCS generally inhibiting cortical excitability, while, in the context of tACS and tRNS, aspects such as stimulation frequency can produce different results. He et al. (2021), using tACS, reported that training improvements when stimulation was delivered at 10 Hz, but not 20 Hz or 40 Hz, lead to improvements. Similarly, Fertonani, Pirulli and Miniussi [57] showed that high-frequency (600–1000 Hz) but not low-frequency (0.1–100 Hz) tRNS increased learning effects with respect to the sham, while Moret, Donato, Nucci, Cona, and Campana [147] showed that offline tRNS cortical modulation in the motor cortex was observed only when the range of stimulation frequencies was large (100 Hz–700 Hz), but not when it was only high (400–700 Hz) or low (100–400 Hz). Of note, some studies reported changes in polarity-specific effects as a function of distance between the electrode and the targeted area. Anodal tDCS increased and cathodal tDCS decreased cortical activation in units close to the electrode in mice studies [89], while the opposite (facilitation after cathodal tDCS and inhibition after anodal tDCS) was observed for units in deeper cortical layers [148].

To sum up, differences between the simple relationship between type of stimulation and measurable effects on the motor cortex vs. the complex effects in the visual cortex can be at least partially explained in terms of anatomy, baseline performance, underlying system activity, stimulus, and stimulation intensity and type [149]. Recent models of tES in the visual cortex take into account features of the stimulation, baseline activity of the visual system and stimulus-driven activity [119], which seem consistent with behavioral results using visual tasks and occipital stimulation (e.g., [67]).

Concerning the **stimulus**, different cortical activations in sensory areas and performance levels are expected for different intensity levels, often subject to large inter-individual differences. Different types of stimuli can selectively engage different areas. For example, simple gratings would maximally engage the early visual cortex and audio–visual stimulation would tap into multisensory integration regions in the parietal lobe, while attentional manipulation can engage prefrontal, cognitive regions as well as perceptual areas. In terms of paradigm, differences in procedures (constant stimuli vs. long adaptive staircase vs. short adaptive staircase) can lead to differences in learning and transfer (e.g., Hung and Seitz, 2014). Concerning the **stimulation**, the right intensity level seems to be crucial in observing positive, rather than disruptive, perceptual effects. Similarly, different protocols seem to affect the targeted populations differently, with some general effects (e.g., anodal tDCS increasing and cathodal tDCS decreasing cortical excitability) and some specific to parameters (e.g., tRNS is mostly effective in boosting learning for a large range of frequencies [147]; anodal tDCS appears to boost learning only when delivered before the training session [72]; tACS enhances learning effects at 10 Hz but not at 20 and 40 Hz [80], etc.). Finally, the targeted location plays a role in the behavioral effects, depending on whether it is directly engaged in the visual task and depending on which subcomponent of the training procedure (i.e., task vs. stimulus component) the targeted area subserves.

## 7. Perspectives on the Combined Use of tES and VPL

We here present a series of observations on these techniques that we hope can guide future studies on this topic.

- Learning is a complex and dynamic process involving low-level, perceptual regions as well as higher-level, cognitive and attentional areas [6,8,31,150]; moreover, multiple mechanisms, acting in series or in parallel [10] underlie learning, potentially resulting in modifications of the functional specialization of cortical areas [151,152]. Our current understanding of brain mechanisms involved in learning conveys an image of higher sophistication than the earlier studies led us to believe. Multiple mechanisms, parallel or serial, are involved. For example, Jing and colleagues [8], using monkey electrophysiology, reported that improvements in a global form detection task were accompanied by parallel neural changes in both sensory and prefrontal areas, which exhibited different time courses within each area as the training progressed. Specifically, stimulus- and task-dependent changes emerged earlier in sensory areas (V4) than in prefrontal areas (ventrolateral prefrontal cortex) and exhibited high specificity for task and target features, while behavioral-related changes followed the opposite pattern, emerging earlier in prefrontal than in sensory areas and exhibiting larger generalization to untrained configurations. Similarly, Shibata and collaborators [10], using human neuroimaging, showed evidence for task- and stimulus-related plasticity, taking place in different regions of the occipital cortex and intraparietal sulcus, following motion-detection training. There is also evidence of different time-specific learning mechanisms. Itthipuripat and colleagues [153], using electrophysiology, suggested that learning has an initial phase dominated by an increase in attentional gain, later replaced by noise reduction mechanisms. Moreover, neuroimaging evidence suggests that VPL is characterized by dissociable neural and functional changes in the visual cortex over time [151,152,154].

Chang and colleagues [151] showed that TMS over the posterior parietal cortex disrupted the extraction of depth cues in noise, but only before participants were trained in a fine-depth discrimination task. Similarly, Chen and colleagues [152] found that inhibiting the activity of MT+ would affect extracting motion direction from noise, but only before motion discrimination training. Similar results have been reported in studies with non-human primates [155,156].

**Figure 2:**
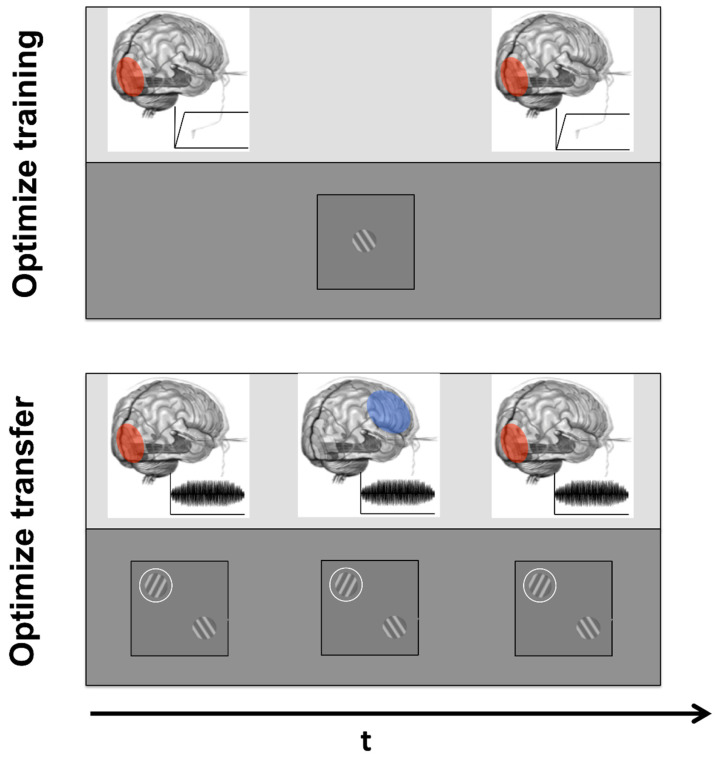
Examples of combined use of brain stimulation and perceptual learning to optimize learning (above) and transfer (below). Above: VPL can benefit from the help of tES to boost both neural plasticity before training (e.g., [72]) and consolidation after training [73]. Below: Having a task that engages both perceptual and attentional mechanisms and a stimulation protocol that targets both low- and high-level cortical regions might lead to larger generalization effects at both perceptual and cognitive levels.

Taken together, these results suggest that multiple brain regions and time-dependent dynamics dominate learning, which could result in changes in systems that are read-out to solve a given visual task. Multiple brain regions involved in learning mean that both multiple possible sites of stimulation and multiple sites can be selectively engaged by accurate task design (see Figure 2). Studies using brain stimulation and VPL should be cognizant of such dynamics and complexities and use our understanding of learning mechanisms from VPL literature to optimize the selection of stimulus, protocols, and target regions of stimulation.

- tES modulates cortical excitability beyond sensory and motor areas. While the majority of tES studies has focused on motor or sensory brain regions, multiple pieces of evidence suggest that tES can modulate cortical excitability in brain regions preferentially involved in higher-level processes implicated in vision such as attention and cognition. Arif and colleagues [157] showed that occipital anodal tDCS has a polarity-dependent effect on the neural oscillations subserving attentional reorientation in adults and that such effects may be related to altered concentrations of GABA within neural networks involved in attentional reorientation. Contò and colleagues [86] showed improvements in behavior and functional connectivity between nodes of the dorsal and ventral attention network when tRNS was delivered over the intraparietal sulci during attentional training. Furthermore, long-term effects of multi-session tRNS have been reported for dorsolateral prefrontal cortex, resulting in a boost in mental arithmetic performances 6 months post-stimulation, which also correlated with an increase in activity within the stimulated area [158].
- Select the stimulation protocol to optimize the behavioral outcome. Different protocols can be used to achieve different results. tRNS seems to be more effective in improving learning rate [57,74] and generalization [81,82,122] when used during training, while anodal tDCS boosts both perceptual performance and learning consolidation when used before [72] or after [73] behavioral sessions, respectively, rather than online. Similar to tRNS, tACS seems to be effective in modulating cortical excitability mainly when used online [159].
- Optimize stimulus and stimulation intensity. Brain stimulation effects on behavior are dependent upon the intensity of the stimulation [67]. The optimal stimulation intensity might in turn depend on the targeted cortical region(s), the task at hand, and the participants’ individual threshold. Additionally, the size of the affected cortical and the current density are dependent on both the stimulation intensity and the size of the electrode [160]. Additionally, there is evidence of non-linear effects involving timing and dosage. Mosayebi-Samani conducted a systematic exploration of the effects of parameters manipulation on tDCS over the motor cortex [161]. The results showed non-linear effects of stimulation intensity and duration, in particular intensities of 1 and 3 mA reduced cortical excitability, while 2 mA increased it. Additionally, 1 and 3 mA stimulation for 15 min induced long-term depotentiation-like plasticity, while on the contrary, 20 min of 2 mA stimulation induced long-term potentiation-like plasticity. Agboada and colleagues (2020) compared a 15 min session of motor cortex anodal tDCS at 1 mA with a 20 min session at 3 mA [162]. When cortical excitability was measured after a single session, both protocols showed a 30 min aftereffect when compared to the sham. When a second session was delivered after a 20 min interval, the aftereffect of the 3 mA protocol lasted 2 h, while that of the 1 mA was still present after 24 h. When the second session was instead delivered 3 h after the first, no increase in cortical excitability was observed for the 3 mA, and only a minor increase was observed for the 1 mA intensity. This once again points toward non-linear effects of the numerous parameters involved in brain stimulation. Importantly, such systematic studies have not yet been conducted in the visual cortex. Further evidence supports intensity-dependent effects in tACS as well. Specifically, Johnson and colleagues (2020), using monkey single-cell recording, showed that tACS-induced modulation, in the form of phase entrainment, and increase in spike frequency, was proportional to the current intensity, with more units exhibiting modulation for higher intensities of stimulation [163]. Similarly, VPL effects are dependent upon the intensity of the stimulus; training too close to the threshold might disrupt transfer of learning [164], while providing a variety of stimulation might prevent sensory habituation [30]. In the context of stochastic resonance, both stimulus and stimulation intensity combine to produce the final behavioral outcome. To optimize such an outcome, one should carefully select and control for both, from choosing the size of the electrodes (and possibly, the electrode configuration montage; see [52,59]) and the current intensity to the experimental paradigm and features of the stimuli, such as size, orientation, contrast, speed, etc.
- Understand the time course and washout of tES to optimize learning consolidation. While brain stimulation is commonly used before or during training to increase cortical excitability and to boost neural plasticity, post-stimulation effects are somehow overlooked. There is evidence of post-stimulation washout effects of tDCS [70], tACS [93] and tRNS [94,165] extending beyond the window of stimulation for over 1 h, which could potentially disrupt some of the learning gain. Thus, promoting consolidation by means of brain stimulation might produce more robust learning. Reis and colleagues [51], and more recently Yang, He and Fang [73], showed that anodal tDCS delivered after motor and visual training, respectively, led to larger learning effects than the sham.

Effective learning needs both plasticity and consolidation (see Figure 2); however, no study thus far investigated the possibility of using brain stimulation to both boost and consolidate learning.

- Individual differences. Both brain stimulation and VPL are sensitive to inter-individual variability [55,166,167,168]. Several factors pertaining to subjective characteristics might play a role in the training effects, with evidence from both VPL and brain stimulation literature showing that anatomy, sex, age, and initial performance/cortical excitability baseline modulate training effects. For example, Kasten and colleagues showed a correlation between individual electric field variability and the modulatory effects of occipital tACS in increasing alpha power [169]. Similarly, Mosayebi-Samani and colleagues addressed observed inter-individual differences in tDCS-induced motor cortex excitability by estimating individual electric fields and anatomy [170]. The results showed that anatomical factors such as electrode-to-cortex distance and cortico-spinal fluid thickness negatively correlated with individual electric fields, which in turn correlated positively with tDCS cortical effects. Chaieb and colleagues [55] reported larger anodal (but not cathodal) tDCS effects in females with respect to male participants, suggesting that brain stimulation might interact with hormonal cycles. Some of the variability can be reduced following stimulation guidelines (e.g., [171]) by collecting a larger set of assessment tasks and individual difference measurements (i.e., questionnaires) and by using more sophisticated statistical models that include mediators and moderators of the effects we observe.
- Understand the limitations of tES. While it is a powerful tool, tES still presents some constraints, mostly of technical nature. It is limited by coarse spatial and temporal resolution, which prevents small structures from being accurately and selectively targeted, and its shallow depth is not ideal for reaching inner structures. Moreover, despite attempts at reducing some of its adverse physical effects [172], some participants might still find it unpleasant, thus affecting compliance. Finally, it is of paramount importance to follow strict safety guidelines [171].

## 8. Open Questions

The relative novelty of this combined approach and the consequent size of the literature are bound to leave several open questions. Here, we list some of these questions.

- Lack of systematic comparison of transfer and training effects across stimulation types. Few studies looked at training effects of different stimulation protocols [57,173], or their ideal onset of stimulation with respect to training sessions [72]; however, no comparison of transfer effects has been conducted, except for studies looking at cognitive/arithmetical abilities [173] or targeting higher-level cortical regions [174]. Crucially, transfer of learning is a more relevant measure of the translational value of a technique when it comes to its rehabilitative application.
- Lack of multi-site stimulation effects on learning. Particularly, electric brain stimulation delivered beyond sensory areas. This might be in part due to the use of ‘local’ and ‘sensory’ frameworks of VPL, which interpret learning effects as a product of neural plasticity changes at the early stages of sensory processing, i.e., sensory areas. However, recent results [11,13,71] and models [6] suggest a more complex scenario in which several regions, including those associated with attention, memory and cognition, can be involved in VPL.
- While VPL and tES have been used to treat mild optical conditions (e.g., myopia, presbyopia) or some visual pathologies of cortical nature (e.g., amblyopia), no study thus far has investigated tES effects in severe retinal pathologies, such as those leading to loss of central vision. For central vision loss following macular degeneration, basic intervention with behavioral paradigms might not be sufficient [36,37,175]. The loss of central vision in MD forces these patients to use a peripheral retinal spot to replace the fovea; thus, any intervention in MD should consider the need for this clinical population not only to improve the detail resolution of their peripheral vision, but also to reroute their oculomotor reference and attentional system toward a peripheral region that must be repurposed to accomplish this feat.

While results in simulated central vision loss seem to suggest that characteristics of oculomotor training could help MD participants develop PRL more rapidly [176,177,178,179,180], brain stimulation might provide a necessary boost to promote larger-scale cortical reorganization. Evidence of significantly larger transfer [122] and training effects in peripheral vision of healthy participants [74] is an encouraging result toward using this technique to improve peripheral vision in MD.

- Lack of follow-up studies to quantify and evaluate long-term effects of tES. Unlike the vast literature on the long-term effects of the use of VPL paradigms alone [3,26,175], studies looking at the lasting effects of tES and VPL together are limited (see Table 1, last column). The few studies that did conduct follow-up tests of training effects are encouraging, suggesting that the learning and transfer gains observed by the end of the training are preserved at least 3 to 6 months after the end of the studies [81,84,122] (however, see [74]), in which a subgroup of participants trained with tRNS and VPL showed lack of long-term effect at 3-month follow up.

Finally, we cannot exclude that our understanding of the effects of tES in improving perception, alone or paired with behavioral training, is at least partially limited by the tendency to publish statistically significant results showing advantage of tES vs. sham, often overlooking null or negative results, which would be rather informative in understanding optimal parameters for the maximization of training effects.

## 9. Closing Remarks

One of the most remarkable human features is the ability to improve in perceptual tasks after repeated practice. A body of work and techniques falling under the umbrella term of perceptual learning has studied this skill for close to 200 years, with the first studies dating back to the mid-XIX century. Early evidence and models of perceptual learning, especially in the visual domain (visual perceptual learning, VPL), suggest that this phenomenon is characterized by learning specificity (meaning that the improvements observed after practice are limited to the stimulus features and task used during training but disappear when learning is tested for different stimulus features or tasks) and finds its neural substrates in early sensory areas. Both VPL and brain stimulation lead to behavioral improvements due to neural plasticity, most likely in early sensory cortex.

Similar to VPL, brain stimulation effects are often discussed in the form of local and anatomically defined changes, often at the level of changes in neuron membrane potential within the confines of the stimulated region.

In the last few years, VPL studies have used concomitant brain stimulation to boost the learning effect, showing improved effects with respect to each of the two techniques used in isolation. This is often taken as evidence that the two techniques affect the same cortical regions and possible mechanisms. However, recent studies in the field of VPL have suggested that the local nature of its effects may be a methodological artifact due to classic studies in the field, which had little to no changes in stimulus and task features. Thus, location specificity might not be a property of VPL but rather a consequence of the way it has been studied. Later studies showed a transfer of learning after manipulations of classic paradigms and new and more refined models of VPL that tried to capture this newfound complexity.

Combining VPL and electric brain stimulation has led to promising results that can pave the way for important clinical applications of this protocol; however, the use of brain stimulation has not yet followed this new understanding of VPL. Evidence of additional complexity of brain stimulation effects on learning and interaction with VPL further suggests a need for a more thorough investigation of all the possible variables involved in these studies, with the goal of developing testable theoretical frameworks. Recent steps in the direction of a more integrative have been proposed, and this understanding appears crucial in moving the field forward [181].

In order for brain stimulation to unlock its full potential when coupled with VPL, time course, loci and type of stimulation should be carefully selected according to the mechanisms of VPL as we now better understand them. Such an approach holds the promise for more effective training paradigms, which could benefit both healthy and clinical populations.

## Figures and Tables

**Table 1:** Overview of PL studies using tES.

Study	Sample Size	Stimulation Type	Stimulation Region	Control	Training Type	Population	Long-Term Effects	Results Supportive of tES + VPL
Fertonani, Pirulli and Miniussi (2011) [57]	14 per stimulation type	Anodal tDCS (a-tDCS), Cathodal tDCS (c-tDCS), High frequency tRNS (hf-tRNS), Low frequency tRNS (lf-tRNS)	Occipital	CZ	Orientation discrimination	Healthy participants	Not tested	Yes
Pirulli, Fertonani and Miniussi (2013) [72]	14 per combination of timing (online vs offline) and stimulation (a-tDCS vs tRNS), 10 for sham	Online and offline(pre) a-tDCS, Online and offline(pre) tRNS	Occipital	Sham	Orientation discrimination	Healthy participants	Not tested	Yes: tRNS better online, tDCS better offline
Campana et al. (2014) [82]	7 stimulation	tRNS	Occipital	No	Flanked contrast detection	Amblyopic patients	Not tested	Yes
Camilleri et al. (2014) [82]	8 stimulation, 8 sham	tRNS	Occipital	Behavioral only	Contrast detection	Myopic patients	Yes, 3 month follow up	Yes
Camilleri et al. (2016) [85]	10 per group (PL + tRNS, Sham, tRNS)	tRNS	Occipital	Sham and tRNS only	Contrast detection	Myopic patients	Not tested	Yes, PL + tRNS bettr than Sham and tRNS alone
Moret et al. (2018) [122]	10 per group	tRNS	Parietal	Sham	Flanked contrast detection	Amblyopic patients	Yes, 6 month follow up	Yes
Contemori et al. (2019) [74]	16 stimulation, 16 sham	tRNS	Occipital	Sham	Crowded letter discrimination	Healthy participants	Absent on 3 months follow up on a subgroup of participants	Yes
Herpich et al. (2019) [84]	Healthy: 9 per group, Patients:3 tRNS, 6 a-tDCS, 2 sham	a-tDCS, tRNS	Occipital, Parietal	Sham, No-stimulation, Active control	Motion direction discrimination	Healthy participants, Cortical blindess patients	Yes, 6 month follow up	Yes for tRNS but not a-tDCS
Contò et al. (2021) [86]	10 per group	tRNS	Parietal, Middle temporal	Sham	Orientation discrimination, Temporal order judgement	Healthy participants	Not tested	Yes, parietal tRNS on orientation discrimination
He et al. (2021) [80]	17–18 per group	10 Hz, 20 Hz, 40 Hz tACS	Occipital, Parietal	Sham	Orientation discrimination	Healthy participants	Not tested	Yes for 10 Hz, no for 20 Hz/40 Hz
Yang, He, Fang (2022) [73]	17 stimulation, 16 sham	Offline (post) a-tDCS	Occipital	Sham	Texture discrimination	Healthy participants	Not tested	Yes

## Data Availability

As this is a perspective paper which reviews current literature, it does not contain new data or involves informed consent from participants.
